# Supraglottic Myxedema Presenting as a Complication of Hypothyroidism: A Case Report

**DOI:** 10.7759/cureus.56903

**Published:** 2024-03-25

**Authors:** Swetha Narayan, Nidhi Bhutra, Sahil Kumar, Jithesh G, Arnab Choudhury, Nitin Kumar, Dr. Ujjawal K Shriwastav, Mukesh Bairwa

**Affiliations:** 1 General Medicine, All India Institute of Medical Sciences, Rishikesh, Rishikesh, IND; 2 Internal Medicine, All India Institute of Medical Sciences, Rishikesh, Rishikesh, IND; 3 Internal Medicine, All India Institute of Medical Sciences, Rishikesh, RISHIKESH, IND

**Keywords:** laryngeal myxedema, airway compromise, levothyroxine therapy, supraglottic myxedema, hypothyroid

## Abstract

Myxedema is a medical emergency with high mortality rates if not treated aggressively. Here, we present a middle-aged female with complaints of generalized body swelling for one year, shortness of breath, hoarseness of voice, neck swelling, and cough for 20 days. The patient was diagnosed to be having severe hypothyroidism with polyserositis. Contrast-enhanced computed tomography (CECT) of the neck and thorax revealed extensive soft tissue edema causing airway narrowing, bilateral pleural effusion, moderate pericardial effusion, and features of atypical pneumonia. The patient was started on levothyroxine and antibiotics as per cultures to which she had initially improved; however, she developed ventilator-associated pneumonia leading to sepsis, acute respiratory distress syndrome followed by refractory type 1 respiratory failure and succumbed.

## Introduction

Myxedema coma is seldom encountered and is a grave complication of hypothyroidism with multiple system dysfunction. Pathologic stressors like infection can overwhelm already distressed homeostatic mechanisms and can precipitate myxedema coma [1]. Even with aggressive management, the mortality rate is highly variable and as high as 60% [2]. Supraglottic myxedema is even rarer, and the precise incidence and prevalence are unknown. It is one of the rarest causes of upper airway obstruction due to mucopolysaccharide deposition in the supraglottic region, including the aryepiglottic folds, false vocal folds, and vocal cords, resulting in local edema [3], which is further compounded by lack of well-established diagnostic criteria and treatment guidelines. Here is the case of supraglottic myxedema as the first presentation of hypothyroidism.

## Case presentation

A 54-year-old female chronic smoker with a history of significant indoor chullah exposure for the last 30 years presented with complaints of generalized body swelling for one year, shortness of breath, change in voice, neck swelling, and cough with minimal expectoration for 20 days. She was evaluated in another hospital, where she was found to have bilateral pleural effusion and pericardial effusion, and was referred to a tertiary-level hospital.

The patient had generalized body swelling from last year, which had increased over the last 20 days. The swelling was present all over the body but was more prominent over the face, legs, and neck. The swelling was associated with a change in voice, which was now of a harsh type. The patient was able to do her daily chores, but from the last 20 days, after the onset of dry cough, her baseline symptoms had increased in severity.

The patient, at the time of admission, had a normal blood pressure of 122/70 mm Hg, a pulse rate of 50 beats per minute, and a respiratory rate of 28 per minute on the nasal prongs at 4 L/min. On general examination, the patient was obese; facial puffiness along with neck swelling was seen, and there was bilateral nonpitting pedal edema. The skin was dry and lusterless. Systemic examination revealed reduced bilateral air entry on the chest, muffled heart sounds, shifting dullness on abdominal examination, and delayed relaxation of all reflexes. The chest x-ray showed pleural effusion with massively enlarged cardiac silhouette.

Based on the above history, clinical examination findings, and evident serositis, a diagnosis of myxedema was suspected. Accordingly, a thyroid profile was sent, which confirmed the diagnosis. The cardiology team performed a 2D echocardiography, which was suggestive of grade 2 diastolic dysfunction, no regional wall motion abnormality, and a global left ventricular ejection fraction of 50%. There was a massive pericardial effusion of 2.1 cm without diastolic collapse of the right ventricle. Thoracocentesis was done, which was transudative in nature. Her acid-base analysis reported a mixed hypoxemic and hypercapnic respiratory failure for which she was intubated and placed on mechanical ventilation. CECT of the neck and thorax was done, which showed extensive edema of the nasopharynx, oropharynx, and hypopharynx and narrowing of the airway. There were also features of atypical pneumonia in the form of ground glass opacities. The patient was admitted and started management in lines of myxedema with high doses of oral levothyroxine (200 mcg once daily) and intravenous steroids (100 mg three times daily). Antibiotics for pneumonia (ceftriaxone and azithromycin) were also administered.

Investigations

Table 1 shows the patient's routine blood investigations.

**Table 1:** Routine blood investigations of the patient g/dL: Grams per deciliter; uL: Microliter; mg/dL: Milligram per deciliter; IU/L: International units per liter; mEq/L: Milliequivalent per liters; SGOT: Serum glutamic-oxaloacetic transaminase; SGPT: Serum glutamic-pyruvic transaminase.

Date	Hemoglobin (g/dl) (normal: 12-16 g/dl)	Total leukocyte count (×10^3^/uL) (normal: 4-11 ×10^3^/uL)	Platelets (10^9^/L) (normal: 150-400×10^9^)	Bilirubin (mg/dL) (normal: 0.1-1.2 mg/dL)	SGOT/SGPT (IU/L) (normal: 10-40 IU/L/29-33 IU/L)	Serum protein (g/dL) (normal: 6-8 g/dL)	Albumin (g/dL) (normal: 3.5-5.5 g/dl)	Serum urea (mg/dL) (normal: 20-40 mg/dL)	Creatinine (mg/dL) (normal: 0.5-1.1 mg/dL)	Sodium (mEq/L) (normal: 136-145 mEq/L)	Potassium (mEq/L) (normal: 3.5-5.2 mEq/L)
7/1/2023	11.5	5.45	110	0.59	143/60	7.1	2.4				
8/1/2023	12	4.48	86	0.34	78/40	5.9	3.2				
9/1/2023								28	0.6	128	4.6
10/1/2023	10.14	1.4	76					27	0.78	125	3.2
11/1/2023	9.7	9.73	59								
12/1/2023	7.1	8.97	53								
13/1/2023	7.4	7.74	54	1.84	45/30	4.7	3.1	35	0.7	129	3.6
14/1/2023	7.7	10.64	68								
15/1/2023	8	13.34	107								
18/1/2023	8	10.3	122	0.83	31/26	4.9	2.9	43	0.8	139	4.1
20/1/2023	6.8	7.06	133					35	0.7	136	3.2
21/1/2023	8.1	5.87	174	0.8	34/26	4.7	2.5				
22/1/2023	8	7.57	150					55	0.8	139	4.5
23/1/2023	7.5	9.9	130	0.9	45			60	1.2	130	5.2

Viral markers were positive for the hepatitis C virus, but the viral load was negative. The thyroid function test revealed an elevated thyroid-stimulating hormone (TSH) level of 37.44 mIU/L (normal: 0.5-5.0 mIU/L) with FT3 and FT4 of 0.54 pg/dL (normal: 2.3-4.1 pg/dL) and 0.33 ng/dL (normal: 0.7-1.9 ng/dL), respectively. Anti-TPO was positive at 656 IU/mL (normal: 0-34 IU/mL). Tropical fever workups for dengue, malaria, and scrub typhus were negative. An electrocardiogram (ECG) showed low-voltage complexes. Ultrasound of the whole abdomen showed only grade 1 fatty liver. Blood, urine, and sputum cultures were sterile. Pleural fluid analysis was transudative in nature, with pleural fluid protein of 0.7 mg/dl and serum protein of 6 mg/dl. Cultures of the pleural fluid were sterile, and the fluid was negative for Mycobacterium tuberculosis as well. A 2D echocardiogram was done, which showed moderate pericardial effusion (with a maximum thickness of 2.9 cm posteriorly and no signs of tamponade physiology). CECT of the neck and thorax showed soft tissue edema involving the nasopharynx, oropharynx, and hypopharynx, causing airway narrowing. Bilateral pleural effusion was observed, measuring 6 cm on the right and 9 cm on the left with moderate pericardial effusion.

**Figure 1:**
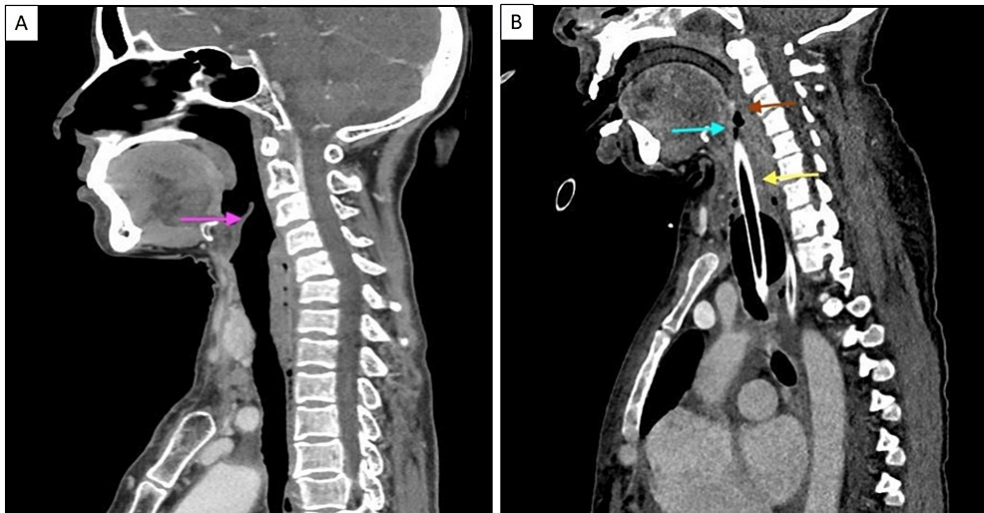
Sagittal CECT image of the face and neck of a normal patient (A) compared to our patient (B) Image A shows a sagittal reformatted CECT image of the face and neck showing normal oropharynx and larynx/hypopharynx with clearly delineated laryngeal cartilage/epiglottis (pink arrow), while image B shows sagittal reformatted CECT image of the face and neck of the patient showing diffuse edema in the oropharynx (red arrow) and larynx/hypopharynx (yellow arrow) causing airway compromise. Laryngeal cartilages/epiglottis (blue arrow) could not be well delineated. The endotracheal tube is in situ. CECT: Contrast-enhanced computed tomography.

Figure 1 shows a sagittal CECT image of a normal patient’s face and neck (Figure 1A) and its comparison to our patient (Figure 1B).

**Figure 2:**
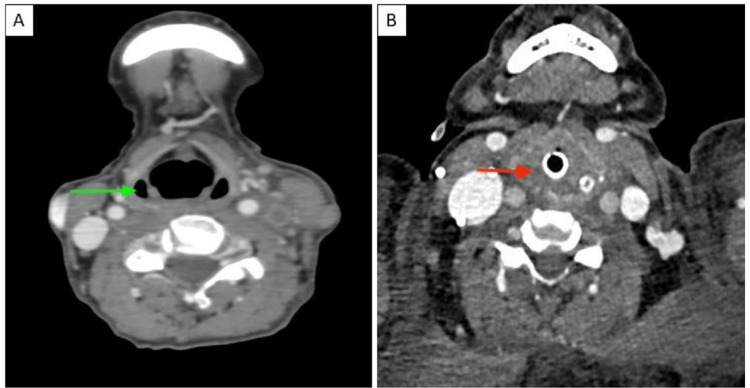
Axial CECT image of a normal patient (A) compared to our patient (B) Image A shows an axial CECT image of the neck showing normal pyriform sinuses (green arrow). In image B, the axial CECT image of the neck of the patient shows effaced pyriform sinuses due to edema in the hypopharynx/larynx (red arrow). The endotracheal tube is in situ. CECT: Contrast-enhanced computed tomography.

Figure 2 shows an axial CECT image showing the normal pyriform sinuses (Figure 2A) compared to our patient (Figure 2B) with effacement of the pyriform sinuses and subcutaneous edema.

Differential diagnosis

The patient had classical hypothyroid facies, bilateral nonpitting pedal edema, and delayed relaxation of all reflexes, which again supported hypothyroidism as a possibility. An elevated TSH level and a positive anti-TPO test confirmed the diagnosis. The patient had a significant chullah exposure history as well as a smoking history. Acute exacerbation of chronic obstructive airway disease due to pneumonia was one of the initial differentials. The patient had a history of bilateral edema feet and worsening breathlessness, so the possibility of acute decompensated heart failure was also considered. However, this was refuted by the fact that edema at the feet was nonpitting from the beginning.

Treatment

The patient was treated with a higher dose of oral levothyroxine 200 mg per day and hydrocortisone 100 mg intravenously thrice daily for a week, after which both were tapered based on the clinical resolution of symptoms. The patient also received ceftriaxone and azithromycin for lower respiratory tract infections. Mechanical ventilation was done for respiratory failure, and gradual weaning was done. An elective tracheostomy was planned because of prolonged intubation.

Outcome

The patient improved clinically and was planned for elective tracheostomy. She developed septic shock due to ventilator-associated pneumonia, for which higher antibiotics (injection colistin 3 MIU IV TDS and meropenem 1 g IV TDS) were administered, and ionotropic support was provided. However, ARDS and refractory type 1 respiratory failure developed, and the patient could not be revived.

## Discussion

Laryngeal involvement is a severe and lethal manifestation of myxedema. The proposed mechanism for laryngeal involvement is the deposition of mucopolysaccharides in the subepithelial space, resulting from aberrant signaling via thyroid hormone receptors within the fibrous connective tissue of the larynx [3]. Besides laryngeal myxedema, other factors contributing to respiratory failure in myxedema include reduced response to hypercapnic and hypoxic respiratory drive, respiratory muscle weakness due to thyroid myopathy, presence of pleural effusion, ascites, obesity, macroglossia, and sleep apnea [4].

We report a case of laryngeal myxedema in a previously undiagnosed hypothyroidism patient presenting with acute shortness of breath, a common finding in most reported cases [4-8]. Our patient also had serositis with pleural and pericardial effusion alongside laryngeal myxedema. Myxedema exacerbation by atypical pneumonia added inflammation to the already swollen larynx. It is crucial to identify and treat the precipitating events of myxedema.

The patient received invasive ventilation for respiratory failure and standard myxedema management with steroids and levothyroxine. Considering the easy availability of oral levothyroxine, a high dose was administered orally. In a literature review by Iftikhar et al. [9], all patients treated for laryngeal myxedema, regardless of the route of levothyroxine administration, had successful outcomes in terms of mortality and discontinuation of mechanical ventilation.

All reported cases of laryngeal myxedema underwent tracheostomies post-intubation due to reasons such as self-extubation, prolonged intubation, and post-extubation stridor [6-11]. Planning early tracheostomies is essential in laryngeal myxedema as all patients will ultimately require it for a variable period.

## Conclusions

Myxedema is a medical emergency that, if left untreated, can lead to high mortality. Although supraglottic involvement in a newly detected treatment-naive hypothyroid patient is rare but should be kept in mind as it can lead to airway compromise. Performing early tracheostomy, treating the precipitant factors, and initiating levothyroxine early are of utmost importance.

## References

[1] Altman KW, Haines GK 3rd, Vakkalanka SK, Keni SP, Kopp PA, Radosevich JA (2003). Identification of thyroid hormone receptors in the human larynx. Laryngoscope.

[2] Jordan RM (1995). Myxedema coma: pathophysiology, therapy, and factors affecting prognosis. Med Clin North Am.

[3] Mathew V, Misgar RA, Ghosh S (2011). Myxedema coma: a new look into an old crisis. J Thyroid Res.

[4] Parving HH, Hansen JM, Nielsen SL, Rossing N, Munck O, Lassen NA (1979). Mechanisms of edema formation in myxedema--increased protein extravasation and relatively slow lymphatic drainage. N Engl J Med.

[5] Benfari G, de Vincentiis M (2005). Postoperative airway obstruction: a complication of a previously undiagnosed hypothyroidism. Otolaryngol Head Neck Surg.

[6] Bicknell PG (1973). Mild hypothyroidism and its effects on the larynx. J Laryngol Otol.

[7] Erwin L (1982). Myxoedema presenting with severe laryngeal obstruction. Postgrad Med J.

[8] Batniji RK, Butehorn HF 3rd, Cevera JJ, Gavin JP, Seymour PE, Parnes SM (2006). Supraglottic myxedema presenting as acute upper airway obstruction. Otolaryngol Head Neck Surg.

[9] Del Prado SRSN, Steinman RA, Munir KM (2017). Supraglottic myxedema: two cases and a review of the literature. AACE Clinical Case Reports.

[10] Iftikhar MH, Raziq FI, Coll P, Dar AY (2021). Laryngeal myxoedema: a literature review of an uncommon complication of hypothyroidism. BMJ Case Rep.

[11] Uzunpinar A (2006). Upper airway obstruction in a patient with severe hypothyroidism presenting as post-extubation stridor. Chest.

